# Antibiotic resistance, virulence factors and genotyping of Uropathogenic *Escherichia coli* strains

**DOI:** 10.1186/s13756-018-0411-4

**Published:** 2018-10-03

**Authors:** Maryam Raeispour, Reza Ranjbar

**Affiliations:** Molecular Biology Research Center, Systems Biology and Poisonings Institute, Baqiyatallah University of Medical Sciences, Tehran, Iran

**Keywords:** *Escherichia coli*, PFGE, UTIs, Virulence genes

## Abstract

**Background:**

The way of treating different types of infectious diseases is really important. Using genotyping method, we can determine the genetic relatedness between the organisms with different resistance profile from different sources. The aim of this study was to determine antibiotic resistance and genotyping of uropathogenic *Escherichia coli* (UPEC) strains using pulsed field gel electrophoresis (PFGE).

**Method:**

*Escherichia coli* (*E. coli*) strains were recovered from the patients with urinary tract infections (UTI) whom admitted in several major hospitals in Tehran. Antibiotic susceptibility testing was done according to CLSI guideline. The present of some virulence factor have been detected using PCR assay. Genotyping of the strains was performed by PFGE and all PFGE profiles were subjected to data processing.

**Result:**

In total, 60 *E. coli* strains were subjected to the study. Most of *E. coli* isolates were resistant to cefepime (100%) and cephalothin (74%) and susceptible to imipenem (100%), vancomycin (100%) and doxycycline (100%). Among the UPEC isolates the prevalence of fimbriae type I (*fimH*), hemolysin (*hlyA*) and aerobactin (*aer*) genes were 89%, 60% and 90%, respectively. The PFGE differentiated *E. coli* strains into 33 different genetic clusters. Majority (30%) of them including PFGE type 11 generated 15 bands, while PFGE type 2 was the lowest (2%) prevalent group with 9 bands.

**Conclusion:**

The result showed that the antibiotic resistance is escalating rapidly. UPEC strains causing infections are more likely to harbor certain virulence genes. Our finding also showed *E. coli* strains isolated under the study were belonged to the diverse clones.

## Background

One of the most common bacterial infections is urinary tract infection (UTI) that accounts for a considerable amount of morbidity and high medical costs and also can lead to significant mortality. While UTI affects men and women it’s most common among women except at early infancy. Approximately 90% of all UTIs in young women are caused by *E. coli* which is a gram-negative, rod-shaped bacterium and a member of the normal intestinal microorganism. Virulence factors are important in severe UTIs [1, 2]. Some of the virulence genes of UPEC strains are aerobactin (*aer*), P fimbriae (*pap*), hemolysin (*hly*), type 1 fimbriae, afimbrial adhesin I (*afa* I), cytotoxic necrotizing factor 1 (*cnf* 1), S fimbriae (*sfa*), adhesins and fimbriae. The other virulence genes that have a role in pathogenecity of organism are: *kpsMT*, *ompT*, *usp*, *iroN*, *iha*, *set 1*, *astA*, group II capsule synthesis; *sfa*/*foc*, S and *F1C*fimbriae; *iutA*, *traT*, serum resistance; and *fimH* [3–5]*.*

In the past recent years, the spectrum and frequency of antimicrobial-resistant UTIs have raised [6, 7]. The resistant patterns of bacteria have been varied by geographical location and by time so periodically testing of antibiotic resistant is really important. *E. coli* strains are the leading causes of serious bacterial infections in health society and very different antibiotic patterns have been reported based on the source [8, 9]. Mobile genetic elements including transposons, plasmids and integrons contribute to lateral transfer of resistance genes in bacteria. *E. coli* can be intrinsically resistant to some special antibiotics and have gens which are responsible for resistance to some of antibiotics such as aminoglycosides, flouroquinolones and β-lactamas [10, 11].

Several studies on various pathotypes of Iranian *E. coli* isolates have done in which a high level of virulence and antibiotic resistance genes have been reported [1, 12–14].

For identification of bacterial infection sources, it is influential to establish relationships between different isolates of bacteria. It helps to determine the contamination sources, gaining insights into the distribution of pathogens, understanding how much pathogens changed over time, to choose the best treatment of diseases, and reducing the risks of antibiotic resistances [15]. Many different typing methods have been established that have been very useful in describing the epidemiology of infectious diseases. The earlier methods were based on phenotypic typing while modern methods have been based on genome components of bacteria [16, 17]. DNA-based approaches have become potentially powerful methods in microbial typing. These techniques consist of analysis of plasmid profiles [18], RFLP (Restriction Fragment Length Polymorphism) [19], Ribotyping [20, 21], MLST (Multi Locus Sequence Typing) [22], VNTR (Variable Number Tandem Repeat) [23, 24], RAPD (Randomly Amplification of Polymorphic DNA) [25, 26], AP-PCR (Arbitrary Pprimed PCR) [27], Rep-PCR (Repetitive extragenic palindromic) [28], ERIC-PCR (Enterobacterial Repetitive Intergenic Consensus) [29, 30], Microarray [5, 31] and PFGE (Pulsed-Field Gel electrophoresis) [32, 33].

The characteristic of typing methods such as discriminatory power, ease of performance, reproducibility, ease of interpretation, and the cost is really important to gain appropriate results. A broad range of methods used to type *E. coli* but PFGE is a commonly used technique for generating DNA fingerprints [28, 34]. PFGE is nominated as the gold standard technique for typing foodborne bacteria [35, 36]. We aimed the current study to determine antibiotic resistance and genotyping of uropathogenic *E. coli* strains isolated from the patients with urinary tract infections in Tehran.

## Method

The uropathogenic *E. coli* isolates were collected from several major hospitals in Tehran during May to November 2016. The bacterial isolates were identified as *E. coli* by standard microbiological and biochemical method. The growth of a single colony with counts> 10^5^ colony forming unit/ml were considered as positive urine cultures. All *E. coli* isolates were inoculated on MacConkey agar then incubated for 24 h at 37 °C. The typical purple colonies were then streaked on Eosin Methylene Blue (EMB) agar plates and were incubated for 20 h at 37 °C. Those colonies with metallic green morphology were subjected to biochemical tests, including hydrogen sulfide, citrate, urease and indol [13].

### Antimicrobial susceptibility testing

Antimicrobial susceptibility was tested by the Kirby-Bauer disk diffusion method. Antimicrobial agents tested were amikacin (30 μg), cephalexin (10 μg), ciprofloxacin (5 μg), cefalothin (30 μg), cefexime (5 μg), cefpodoxime (10 μg), cephazolin (30 μg), cefepime (30 μg), doxycycline (10 μg), nitrofurantoin (30 μg), gentamycine (10 μg), nalidixic acid (30 μg), norfloxacin (5 μg), cotrimoxazole (30 μg), tetracycline (30 μg), imipenem (10 μg), and vancomycin (30 μg). These antibiotics belong to β-lactamas, glycopeptides, aminoglycosides, quinolones and tetracycline classes [10]. The resistance to above mentioned antibiotics was determined according to the breakpoint proposed by CLSI [37]. For quality-control *E. coli* ATCC®25,922™ was used.

### Detection of virulence factor

Genomic DNA of UPEC isolates were extracted using DNA extraction kit (AccuPrep® Genomic DNA Extraction Kit, Bioneer, South Korea), after preparation cultured cells with PBS buffer. Binding buffer (GC) was mixed with the samples and incubated for 10 min at 60 °C then washing buffers were added and samples were spined according to the manufacturer’s instructions. Then elution buffer was added to the genomic DNA and stored at − 20 °C.

All UTI isolates were screened for carriage of *fimH*, *hlyA* and *aer* virulence factors. PCR assays were used to reveal the prevalence of these virulence genes using specific primers. The amplification reaction were carried out in a final volume of 25 μl containing 200 μM of deoxynucleotide triphosphates (dNTPs), 2.5 μl of 10X PCR buffer, 0.7 mg/μl MgCl_2_, 0.6 units of Taq polymerase, 10 pmol of each primer, and 2 μl of sample DNA. The PCR products were analyzed with gel electrophoresis on 2% agarose, followed by staining with EtBr solution after 1 h under 80v and visualized using an ultraviolet (UV) transilluminator. Primer sequences and PCR machine conditions are shown in Table 1.

**Table 1:** Primers and cycling conditions for the PCR assays

Gene	Primer sequence	Size (bp)	Cycling condition	Reference
*hlyA*	F: AACAAGGATAAGCACTGTTCTGGCT R: ACCATATAAGCGGTCATTCCCGTCA	1177	1 cycle of 95 °C for 5 min;30 cycles of 94 °C for 1 min, 64 °C for 1 min, 72 °C for 1 min; 72 °C for 8 min final extension	[58]
*FimH*	F:GAGAAGAGGTTTGATTTAACTTATTG R: AGAGCCGCTGTAGAACTGAGG	559	1 cycle of 95 °C for 5 min;30 cycles of 94 °C for 1 min, 60 °C for 1 min, 72 °C for 1 min; 72 °C for 8 min final extension	[60]
*aer*	F: GCTGGGCAGCAAACTGATAACTCTC R: CATCAAGCTGTTTGTTCGTCCGCCG	602	1 cycle of 95 °C for 5 min;30 cycles of 94 °C for 1 min, 62 °C for 1 min, 72 °C for 1 min; 72 °C for 8 min final extension	[58]

### Pulsed field gel electrophoresis procedure

All the PFGE steps were accomplished following the CDC-standardized procedure used by all PulsedNet laboratories with some changes [38]. Colonies from an overnight culture were suspended in TE buffer (5 mmol/liter Tris-HCL [pH 8.0] 1 M, 10 mmol/liter EDTA 0.5 M) at a wavelength of 600 nm (OD = 0.8–1.2). Then proteinase K, 20 mg/ml added to suspension and mixed with sodium dodecyl sulphate and 1% melted SeaKem Gold agarose and pipette into plug moulds and left at 4 °C. After solidation, each plug was transferred to a falcon containing 5 ml cell lysis buffers (2.5 ml Tris-HCL [pH 8.0] 1 M, 5 ml EDTA 0.5 M, 5 ml Sarcosyl 10%) and proteinase K and then incubate overnight at 52 °C. The plugs were washed three times for 30 min in sterile distilled water and twice for 30 min in TE buffer at 52 °C on an orbital shaker. 2 mm of *E. coli* colonies plugs were digested overnight with 50 U of *Xba*І restriction enzyme. And *Salmonella* Braenderup H2812 was used as size standard which after digestion with *Xba*І generates fragments ranging from 20.5 to 1135 kb and cover the fragment ranges generated by *E. coli* strains [39].

After restriction digestion the plugs were placed in refrigerator for 30 min. PFGE was performed with the CHEF DR-III (Bio-Rad Laboratories) system using a 1% Ultra pure agarose gel in 2 l 0.5 TBE (Tris-base, EDTA, boric acid). The electrophoresis condition were set as follows: initial switch time, 2.2 s, final switch time, 54.2 s, run time 20 h, include angle 120°, gradient, 6 V/cm, temperature, 14 °C. The gel was stained for 30 min with EthBr, 0.5 mg/ml and then the fingerprinting profile was observed by Uvitec system by illuminated UV wave to the gel.. The PFGE profiles were compared using In silico stimulation of molecular experiment with a dice similarity coefficient and UPGMA analysis to create the dendrogram. To analyze genetic relatedness, cut off line at 85% was considered [38–41].

## Results

### Bacterial isolates and antibiotic susceptibility testing

Over 6-month period, a total of 60 *E. coli* strains were isolated and subjected to this study. Distribution of the isolates based on patient’s gender and location in Tehran is shown in Table 2. As it is represented in Fig. 3, these 60 isolates identified to be multidrug resistant (MDR). The different prevalence of each antibiotic resistance was as follows: cefepime (100%), cefalothin (74%), cefpodexime (67%), nalidixic acid (63%), cotrimoxazole (54%), cefixime (50%), cephazolin (50%), tetracycline (50%), norfloxacin (43%), ciprofloxacin (34%), cephalexin (30%), gentamycin (19%), nitrofurantoin (10%), amikacin (8%), doxycycline (0%), imipenem (0%), and vancomycin (0%). (Table. 2).

**Table 2:** Date of isolation, age, sex and location of patients admitted to the hospital; isolates divided in different pulsotype

Pulsotype	Isolation(NO.)	Sex	Age(year)	Patient location	Date of isolation
1	13u	F	27	North	30/05/2016
	8u	F	79	North	18/05/2016
	84u	F	52	Northeast	26/05/2016
	94u	F	20	Northeast	18/07/2016
2	30u	F	41	West	22/11/2016
3	4u	F	24	Center	25/11/2016
4	6u	F	63	West	10/10/2016
5	21u	M	55	West	06/08/2016
	23u	M	62	West	23/08/2016
6	7u	F	26	North	11/07/2016
7	28u	F	52	Center	14/09/2016
	9u	F	87	Center	23/10/2016
8	59u	F	34	East	18/05/2016
	73u	F	56	East	13/10/2016
9	72u	F	78	North	18/09/2016
10	100u	F	74	East	06/08/2016
	19u	M	71	Northeast	10/08/2016
11	71u	M	68	Center	18/05/2016
12	10u	M	47	Northwest	03/06/2016
	5u	M	56	Northwest	01/07/2016
	82u	F	48	Northwest	29/06/2016
	24u	F	39	North	03/07/2016
	31u	F	72	North	03/07/2016
13	12u	F	68	North	05/09/2016
	32u	M	70	West	12/09/2016
	11u	F	29	West	03/09/2016
14	62u	M	82	East	28/10/2016
	81u	M	74	Northeast	20/10/2016
15	58u	M	68	North	16/11/2016
	60u	F	50	North	11/11/2016
16	70u	F	73	Center	11/07/2016
17	79u	F	59	Northwest	18/07/2016
18	83u	F	54	Northwest	26/05/2016
19	20u	M	71	Northwest	18/09/2016
	89u	M	67	North	30/07/2016
	92u	F	49	Center	22/08/2016
	97u	F	59	Center	10/08/2016
20	14u	M	64	Northeast	06/08/2016
21	80u	F	40	East	11/10/2016
22	16u	F	24	Northeast	28/10/2016
	18u	M	66	Northeast	18/10/2016
	74u	F	27	Northeast	23/09/2016
	76u	F	46	West	22/10/2016
	25u	F	38	West	18/10/2016
23	86u	M	53	West	24/05/2016
	87u	F	36	North	26/05/2016
	93u	M	83	North	26/05/2016
24	15u	F	27	Center	25/08/2016
25	99u	F	70	Center	14/08/2016
26	17u	F	36	West	25/09/2016
	26u	M	68	West	23/09/2016
27	78u	F	24	Center	11/10/2016
28	2u	F	77	Northeast	25/05/2016
	3u	M	68	Northeast	17/08/2016
29	35u	F	74	Center	15/07/2016
30	88u	F	31	Center	11/07/2016
31	61u	F	26	North	24/06/2016
	98u	F	47	North	13/06/2016
32	77u	F	33	Center	11/10/2016
33	85u	F	33	West	30/05/2016

### PCR assay

**Fig. 1:**
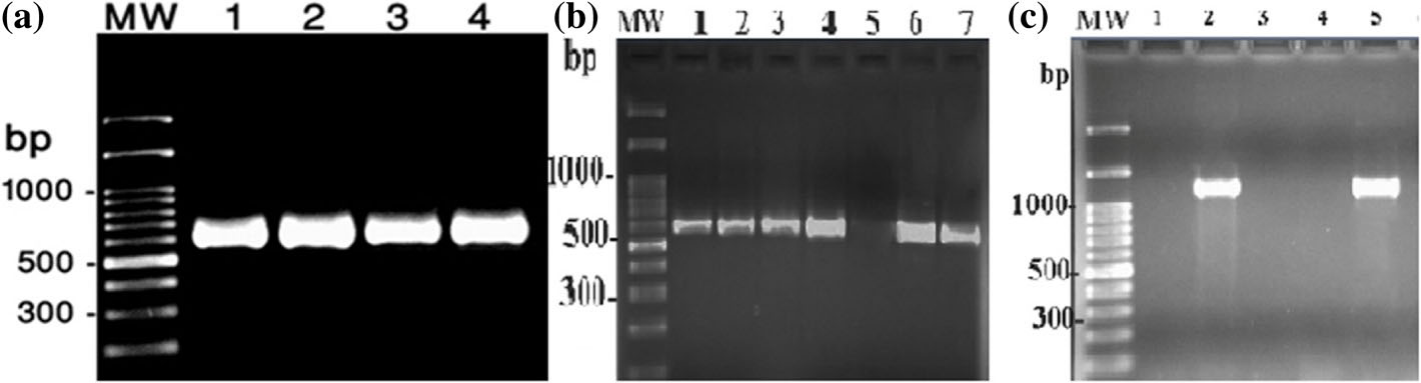
Detection of *aer* (**a**), *hly* A (**b**) and *fim H* (**c**) gens in some representative *E. coli* isolates

The PCR amplified successfully *aer*, *fimH* and *hlyA* genes with 602 bp, 559 bp and 1177 bp amplicons respectively. The PCR results of some representative isolates are shown in Fig. 1. Among 60 UPEC isolates, *aer* was the most prevalent virulence gene (*n* = 54, 90%) followed by *fimH* (*n* = 53, 89%) and *hlyA* (*n* = 36, 60%). All of the isolates carried at least one virulence gene.

### Pulsed field gel electrophoresis

**Fig. 2:**
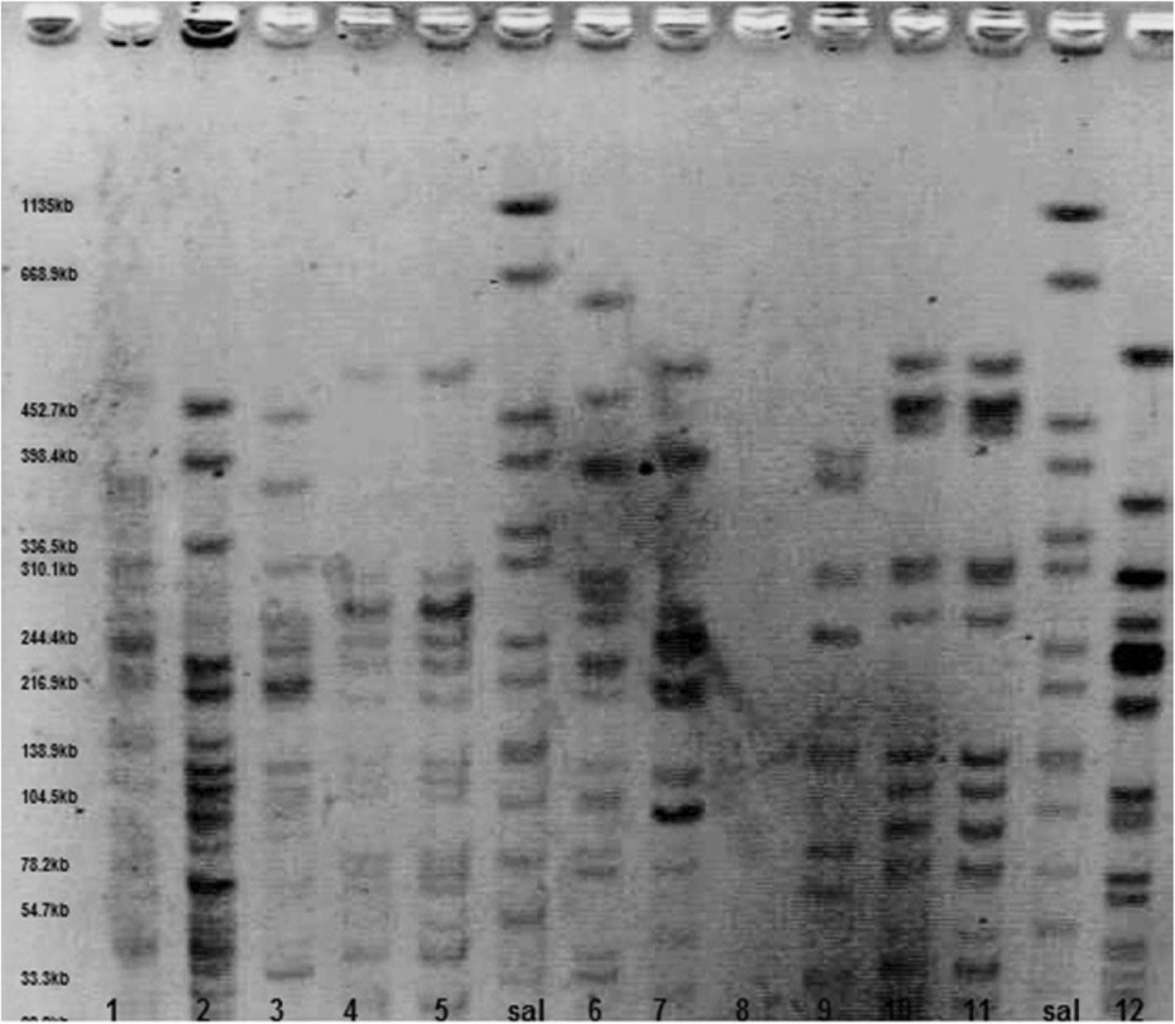
Representative PFEG profiles of some *E. coli* strains; *Salmonella* Braenderup used as molecular size standard (1135–28.8 kb)

**Fig. 3:**
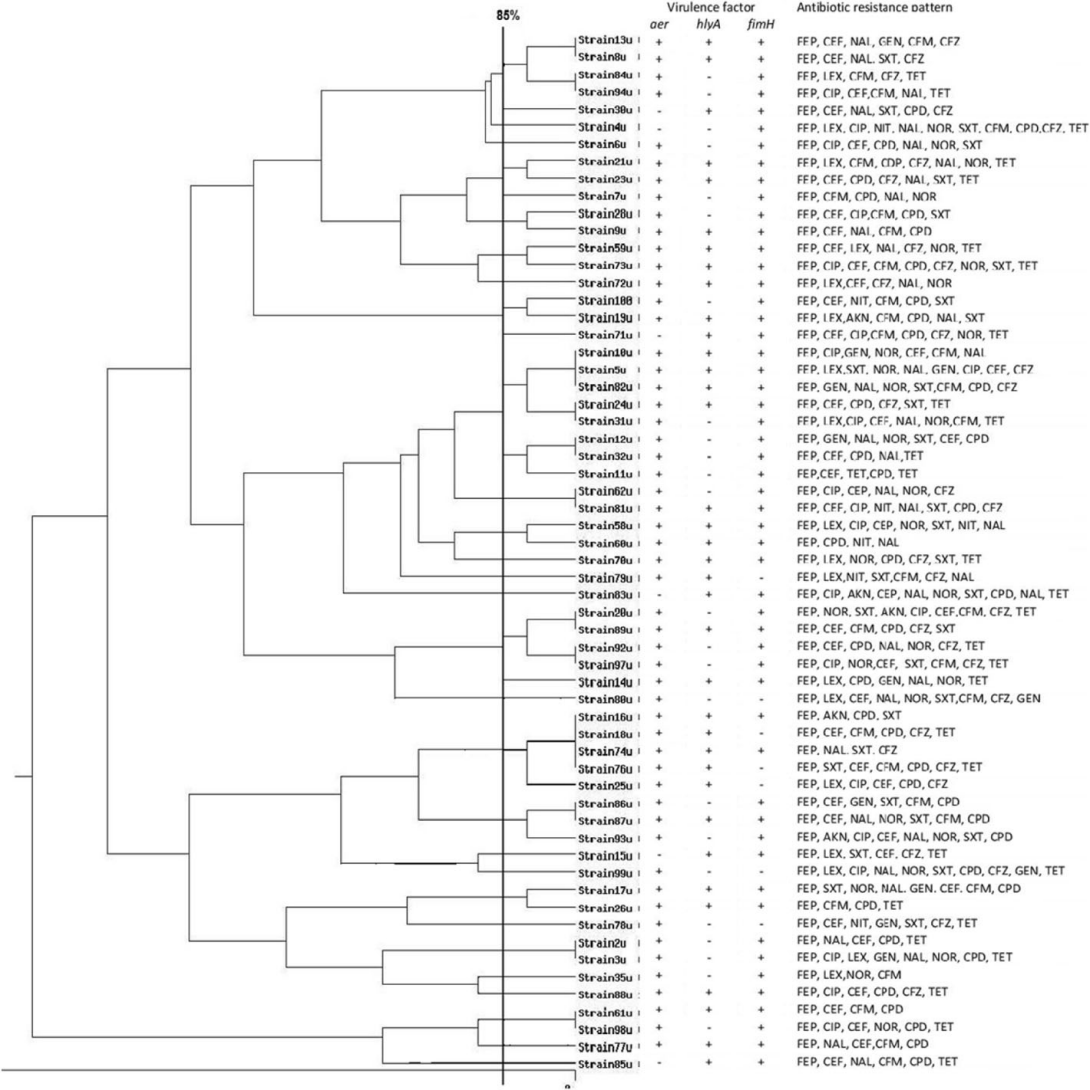
Dendrogram of 60 *E. coli* isolates based on PFGE patterns after digestion with enzyme *Xba*І associated with present of virulence factors (*fimH*, *hlyA* and *aer*) and antibiotic resistance pattern; AKN, amikacin; *LEX*, cephalexin; *CIP*, ciprofloxacin; *CEF*, cefexime; *CDP*, cefpodexim; *CFZ*, cephazolin; *FEP*, cefepime; *NIT*, nitrofurantoin; *GEN*, gentamycin; *NAL*, nalidixic acid; *NOR*, norfloxacin; *SXT*, cotrimoxazole; *TET*, tetracyclin

After observing PFGE profiles fingerprinting (Fig. 2), clustering analysis performed and 33 PFGE pulsotypes were detected for *E. coli* isolates as seen in the dandrogram (Fig. 3). Eighteen clusters including (pulsotypes 2,3,9,11,16,17 and 21) were consist of only one strain, 9 clusters including (pulsotypes 7,14,29 and 31) consist of 2 strains, 2 clusters including (pulsotypes13and 26) consist of 3 strains, 2 clusters including (pulsotypes1 and 19) consist of 4 strains, and 2 clusters including (pulsotypes 12 and 22) consist of 5 strains. Distribution of the pulsutypes is shown in Table 2.

## Discussion

*E. coli* is considered as the cause of 80–90% of UTIs that today is one of the most common bacterial infections [42]. Because of unreasonable use of antibiotics, the bacterial resistance has been raised. In this study, we reported a high value of multidrug resistance among the uropathogenic *E. coli* strains. Resistance to cefepime was very high (100%) and after that the strains were resistant to cefalothin (74%) and cefpodoxime (67%). Also high sensitivity to imipenem, vancomycin and doxycycline (100%), amikacin (92%) and nitrofurantoin (90%) have been observed. High levels of susceptibility to imipenem, amikacin, nitrofurantoin and also high levels of resistance to tetracycline and ampicilline have been reported in other studies in Iran [6, 13].

Our results in some cases are consistent with those reported by Niranjan and Malini. They evaluated antibiotic resistance of 119 *E. coli* isolated from UTI patients. The isolates were resistant to ampicillin (88.4%), amoxicillin (74.4%), norfloxacin (74.2%), ceftiriaxone (71.4%) and sensitive to amikacin (82.6%), nitrofurantoin (82.1%) and imipenem (98.9%) [43].

No resistance to vancomycin, imipenem and doxycycline was observed among the studied isolates. In previous studies a high sensitivity to imipenem has been also reported [43–46]. These antibiotics seems to be a good choice for the treatment of UTI caused by *E. coli* but it should be considered that unlimited use of these antibiotics can gradually lead to increasing antibiotic resistant. Shakya, did a research on antibiotic resistance of *E. coli* strains isolated from Indian children. The results showed that the strains have been resistant to nalidixic acid (45%), tetracycline (37%), ampicillin (37%), trimethoprim/sulfamethoxazole (29%), amoxicillin/clavulanic acid (29%), imipenem (0.0%) [45].

Susceptibility to amikacin in our study was high as it was in studies carried out in other parts of the world [43, 46, 47]. However, the resistance to tetracycline in our study was 50% while in another study it was reported to be 26% [48].

In the present study, it has been shown that resistant to gentamicin (19%) and nitrofurantoein (10%) was low among *E. coli* isolates and it was partially similar to the study reported by Adib and his colleagues. They showed that 54.16% of the isolates were resistant to nalidixic acid, 36.45% to gentamycin, 71% to cefazolin, 29.18% to ciprofloxacin, 14.58% to cefepime, 6.25% to nitrofurantoin, and 0.0% to imipenem [44]. However in other studies the resistance against gentamycin for *E.coli* isolates in Iran was about 93% and 62% [12, 14]. Villar et al. reported 99% and 98 susceptibility to imipenem and amikacin respectively for their uropathogenic *E. coli* strains that is consistent with our results [46].

In this study, we have shown the presence of *aer*, *fimH* and *hylA* genes in UPEC isolates (Fig. 3). Almost all of the isolates harbored *aer* gene, which is responsible for obtaining iron. The frequency of *aer* gene in our study is higher than recent studies [49, 50].One of the secreted toxine factors in *E. coli* strains is *hlyA*, which implicated in tissue damage and dysfunction of local immune responses. This study showed a higher prevalence of *hlyA* among UPEC strains in contrast to other studies [49, 50]. Of note, *FimH* is highly conserved and extremely common among *E. coli* isolates. *FimH* mediates UPEC adherence to the urothelium cells, and helps the formation of intracellular bacterial biofilms [51]. The distribution of *fimH* gene in our strains is in agreement with other published data [1, 49, 52].

Information about the source of the infections and relatedness between bacterial agents is helpful for preventing measures and choosing the best treatment [35]. In recent studies PFGE, as a gold standard method, has been used for DNA fingerprinting and epidemiologic studies successfully. PFGE is a high discriminatory and reproducible typing method that is used in CDC laboratories [34, 53]. This study provides more information on the distribution of urinary isolates of *E. coli* strains. We applied PFGE technique for separating fragments of DNA chromosome digested using XbaІ restriction enzyme. This enzyme has been proved to be more powerful and it is the most common used restriction enzyme for UPEC outbreaks [54–56], and was successfully used in this study to discriminate between the isolates from the UTI patients in some major hospital in Tehran. By using XbaІ enzyme the analyzed isolates generated an exclusive profile with the number of DNA bands between 9 and15 and the bands from 33.3kbp to 1135kpb, while other studies have shown different number of bands and different molecular weights. Anvarinejad et al. reported 9 to 16 DNA bands with molecular size of 2 to 660kbp in molecular typing of *E. coli* isolates from patients with cyctitis and pyelonephritis [57]. Dong et al. reported 15–20 distinct bands in genotyping of Shiga toxin produced by *E. coli* isolates [41]. Ejrnaes et al. reported 15–20 bands with 50kbp to 1200kbp molecular sizes by typing UPEC strains [8].

In the current study, based on drown dendrogram 33 clusters with 85% similarity were found among 60 isolates. The strains with 12 and 15 bands had the highest percentage of 30% and 25% respectively, and the lowest percentage was for the strains with the 9 (2%). The pattern No. 10 with 12 bands and No. 12 with 15 bands were repeated more than other patterns in the present study (Fig. 2). By considering the distinct pulsotypes obtained in this study it seems that there is genetic heterogeneity of *E. coli* in the region. Of note, between 60 MDR isolates collected from 6 different locations in Tehran 84% of isolates belonged to the Center had specific types and 16% of isolates of North and Northwest had similar PFGE patterns, while in Northeast only 30% of isolates had specific patterns and other types were also found in other locations during 6 month of study. There was no defined pattern between the time of collection and PFGE profiles of *E. coli* isolates. Patrick and Padman investigated the efficacy of PFGE in a study of extended spectrum beta lactamase (ESBL) enzymes produced by pathogen (*Escherichia coli* and *klebsiella* pneumonia) [53]. Miyuki and his colleagues used PFGE-CHEF for epidemiological study of *E. coli* O157: H7 isolates collected from 1997 to 2000 in Northern Ireland and compared the results to phage typing analysis. They reported that PFGE-CHEF typing proved to have a greater ability to distinguish *E. coli* O157 isolates, while phage typing has been shown to be less efficient [58]. Jones et al. used PFGE for determining the presence of *E. coli* O_157_ and *Salmonella* isolates in an outbreak and concluded that 20% of clusters were attributed to the same source [59]. Xiaoli et al. in China did a study about the genotypic characteristic of multidrug-resistance UTIs *E. coli* by PFGE. Forty PFGE types were observed for 51 MDR *E. coli* isolates at the cut off value of 85% [40].

Considering the high diversity of genetic relatedness between our MDR isolates there was not special pattern for antibiotic resistant. However, we found that isolates belonging to pulsotype 3 were resistant to 65% of antibiotics, while isolates in pulsotype 29 were resistant to 23% of antibiotics.

## Conclusion

The present study demonstrates that considering the high number of *E. coli* types in the previous studies our conclusions are not surprising. The PFGE applied in the study was shown to be more powerful. PFGE is an appropriate choice for identifying the sources, clonal relatedness and spread of *E. coli* isolates in the hospitals. The result showed that the antibiotic resistance among uropathogenic *E. coli* under study is escalating rapidly. The PFGE types 3, 18, 25 that consist of 5% of isolates were more associated with antimicrobial resistant. Despite some exception such as PFGE type 1 and 22, most of the isolates in the same type followed similar virulence factors pattern. The information obtained from the similarity of the pulsotypes among isolates can help physicians to understand antibiotic resistant patterns of the different isolates from different sources and have a correct choice in prescribing antibiotic medicine.
